# A novel case of testicular metastasis from prostate cancer mimicking epididymo-orchitis despite suppressed PSA

**DOI:** 10.1016/j.eucr.2026.103457

**Published:** 2026-04-22

**Authors:** Alison Hird, Tomisin Omogbehin, Lauren Matthews, Shiv Pandian

**Affiliations:** a North Middlesex University Hospital, Sterling Way, London, N18 1QX, UK; b Royal Free Hospital, Pond Street, London, NW3 2QG, UK

**Keywords:** Prostate cancer, Testicular metastasis, Epididymo-orchitis

## Abstract

Testicular metastasis is an uncommon complication of prostate cancer and therefore the incidence is poorly understood with the literature citing a range of incidence from 0.18% to 4%. This case report looks at a novel case of prostate cancer with testicular metastasis following good response to treatment for primary prostate cancer. The testicular lesion was found incidentally on surveillance PSMA PET CT, investigated with ultrasound and initially identified as epididymo-orchitis. The testicular lesion was later discovered on repeat imaging to be malignant in origin. Following orchidectomy it was confirmed histologically that the testicular mass was prostate in origin.

## 1 Introduction

Prostate cancer is a major cause of cancer morbidity and mortality in males worldwide. In the UK, prostate cancer is the second most common cause of cancer death in males and accounts for 14% of all male cancer deaths. Prostate cancer routinely metastasises to bone, lung, liver, pleura, and adrenals.^1^ Metastasis to the testes from primary prostate cancer is rare and study is mostly limited to case reports 2, 3, 4, 5, 6. Therefore, the incidence is not well understood and has a high range of variability; further study is necessary to gain better understanding of the significance of this topic. The following case report highlights the importance of considering testicular metastasis as a differential in a patient with known prostate cancer. The patient presented in this case report had a missed testicular metastasis following initial successful PSA suppression from oncological treatment. Although the mass in the testes was found on PSMA PET CT and was investigated with ultrasound, it was treated as epididymo-orchitis, and only later discovered to be a metastasis.

## 2 Case report

A 77yr old gentleman attended A&E with penile pain due to catheter associated trauma. The catheter had been inserted a month prior, due to urinary retention while he was out of the country. On subsequent investigation he was found to have an enlarged prostate on ultrasound renal tract and an elevated prostate specific antigen (PSA) of 59.1 ng/ml. He subsequently had an MRI prostate which revealed a prostate volume of 130 cc, PSA density of 0.11 and a PIRADS 5 left posterior peripheral zone lesion, with bilateral seminal vesicles involvement and possible pelvic sidewall invasion. Preliminary staging of T4 N1 Mx prostate cancer was made after MDT discussion. He had a trans-perineal peripheral zone prostate biopsy and a transurethral resection of the prostate (TURP). The histology from prostate biopsy showed prostate acinar adenocarcinoma with involvement of 3/7 cores on the right side and 7/9 cores on the left side: maximum tumour length 12mm. The tumour was graded Gleason 5 + 5, with areas of Gleason 4 + 3 and Gleason 4 + 5 also present.

Following histo-pathological and further radiology review, diagnosis of T4 locally advanced adenocarcinoma of the prostate involving left pelvic wall and left levator ani muscle, N1 with pelvic lymph nodes and potential M1 subpleural nodules was made. He competed a four week course of Bicalutamide and then commenced on LHRH analogue injections (Leuprorelin). Chemotherapy and radiotherapy were initially offered but started later due to patient choice. He completed six cycles of Docetaxel followed by radical radiotherapy to prostate and bilateral pelvic lymph nodes. Post treatment MRI prostate showed a reduction in size of prostate tumour and PSA of <0.1 ng/ml, indicating good response to treatment.

He was followed up with an annual PSMA PET CT which found a 1.7cm complex lesion in the upper pole of the left kidney, and an asymmetrical, heterogenous intense uptake in the left scrotum. His renal lesion was discussed in the MDT and a decision was made for watchful waiting. Following the PSMA PET CT, he had an USS testis, which showed left moderate hydrocele, enlarged left epididymis, heterogenous left testis with cystic feature and increased doppler colour vascular flow consistent with epididymo-orchitis. The right testis was reported as normal. His case was discussed in the MDT, and it was decided that the testicular pathology was due to epididymo-orchitis which was additionally supported by the patient's symptoms of testicular pain and dysuria. He was treated with antibiotics and his testicular tumour markers were checked and found to be negative.

The next annual follow-up PSMA PET CT found retroperitoneal lymphadenopathy as well as appearances of skeletal deposits in T8, left 8th rib and right inferior ischiopubic ramis which appeared to be metastatic in nature. There was persistent intense activity in the left renal lesion, as well as in the large mixed/cystic lesion of the left scrotum. At this time, his PSA rose to 9.03 ng/mL, and he was commenced on maximum androgen blockade (MAB) with Bicalutamide and later started on the novel anti-androgen Abiraterone. His PSA then fell to below 0.1 ng/mL, following treatment.

**Fig. 1:**
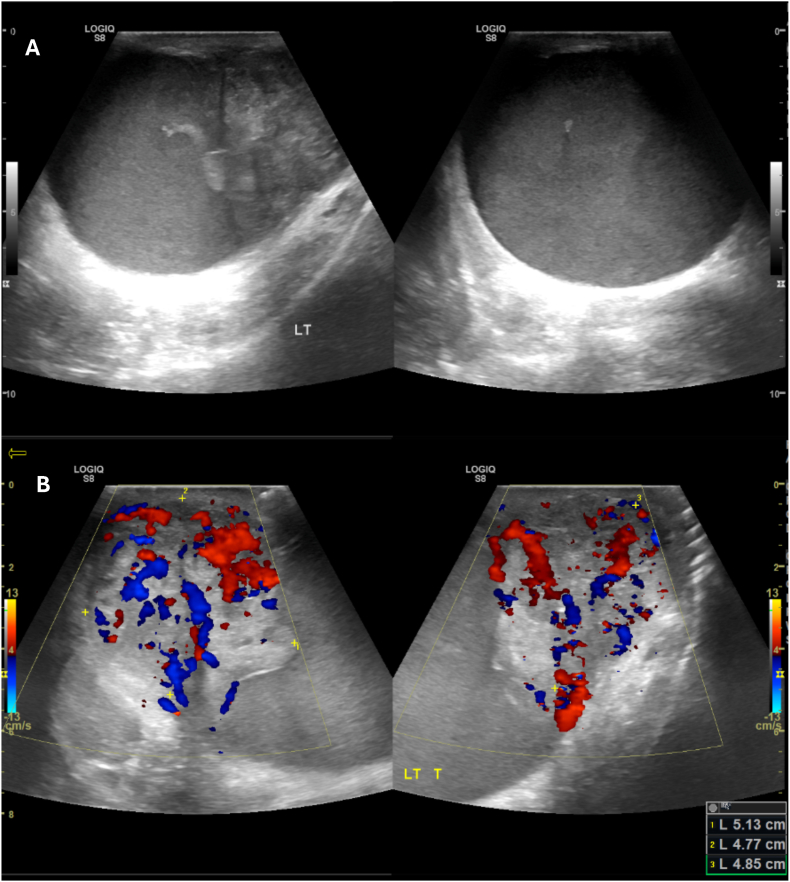
**Ultrasound Testes.** Panel 1A: Ultrasound of left testes showing solid tumour and lack of normal testicular architecture. Panel 2A: Ultrasound of left testes with blood flow overlay showing increased blood flow.

Following his repeat PSMA PET CT, he had a subsequent ultrasound testis [Fig. 1A; Fig. 1B] which reported that the left testicle was barely seen and there was a large irregular mass measuring 5.1x4.8x4.9cm. Patient was discussed in the MDT and the conclusion was that the left testis mass was highly suspicious of malignancy and a left radical inguinal orchidectomy was planned.

**Fig. 2:**
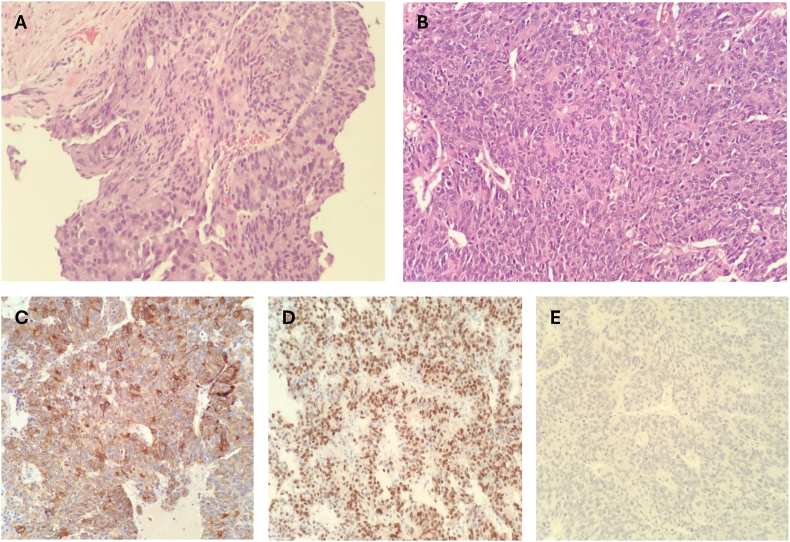
**Histology.** Panel 2A: Prostate core from left base shows prostate acinar adenocarcinoma, Gleason score 5 + 5. Panel 2B: Section from left radical orchidectomy shows metastatic prostate adenocarcinoma. Panel 2C: MNF116 stain (epithelial marker) is positive in tumour cells. Panel 2D: NKX3.1 stain (prostate marker) is positive in tumour cells. Panel 2E: SALL4 (testicular germ cell tumour marker) is negative in tumour cells.

Left radical orchidectomy was performed through inguinal approach and the left testis with surrounding hydrocele sac and distal spermatic cord were removed. The specimen was sent for histopathology, which showed almost complete replacement of the testicular parenchyma by a partly necrotic tumour with invasion into the epididymis and fat at the root of the cord. The tumour was composed of solid sheets of malignant cells with pleomorphic nuclei containing prominent nucleoli and no glandular differentiation. This morphology matched that seen in the areas of prostate adenocarcinoma Gleason 5 + 5, in the previous prostate biopsies [Fig. 2A; Fig. 2B].

On immunohistochemistry, the tumour cells were positive for prostate markers (NKX3.1 and PSA) as well as an epithelial marker (MNF116) [Fig. 2C: Fig. 2D]. The tumour cells were negative for germ cell markers (SALL4, OCT3/4, CD30 and CD117) and sex cord stromal markers (inhibin and melan A) [Fig. 2E]. Together these findings confirmed metastatic prostate adenocarcinoma. It was also noted that there was no morphological or immunohistochemical evidence of neuroendocrine differentiation.

## 3 Discussion

Testicular metastasis is a rare, yet important differential for testicular pathology in a patient with a known cancer. Leukaemia and lymphoma have been identified as the most common cause of testicular metastasis^6^ with prostate being the most common solid tumour cause of testicular metastasis.^7^ Due to the rarity of testicular metastasis from prostate cancer the incidence is not well understood and reported figures range significantly from 0.18% to 4%.^5^^,^^6^^,^^8^^,^^9^ Contemporary hormonal prostate cancer treatment has reduced the need for orchidectomy; therefore, it is probable that cases of metastasis to the testes are being under reported.

Despite the proximity of the male genitourinary organs, prostate cancer is much less likely to metastasize to the testes, than other organs. The main route of metastasis of prostate cancer is thought to be via the prostatic veins to the spine, as well as hematogenous spread though the vena cava leading to bone metastasis as well as metastasis to the lung, liver, pleura and adrenals.^1^ One theory for the low incidence of testicular metastasis is thought to be due to the relatively low temperatures in the scrotum limiting proliferation of tumour.^5^ Additionally, it is thought that the blood-testis barrier protects the developing germ cells with anatomical, physiological and immunological barriers.^10^ Although proposed protective mechanisms to prevent metastasis to the testes have been studied metastasis still occurs. Suggested pathophysiological mechanisms for testicular metastasis of prostate cancer from the literature includes arterial embolism and retrograde venous extension, as well as embolism through the spermatic vein.^3^^,^^6^

This case highlights the importance of the clinical correlation between testicular pathology and primary prostate cancer. The patient described in this report had an excellent response to initial oncological treatment with his PSA falling to below 0.1 ng/mL. Therefore, the suspicion of a new metastasis was reduced and likely why other pathologies were more closely considered when the patient had an increased uptake in the left scrotum on his annual PSMA PET CT. Additionally, the initial ultrasound of the testes following the incidental finding on PSMA PET CT showed no concerning features of malignancy and was reported as likely epididymo-orchitis. Only after a repeat PSMA PET CT the following year, showed persistence of the increased uptake in the left scrotum, was the left testis imaged again with ultrasound. The repeat USS testes more clearly revealed a malignant picture. This was also accompanied by a significant rise in PSA from subclinical levels to 9.03 ng/mL further raising suspicion of metastatic spread. Perhaps repeat USS testes monitoring response to treatment for epididymo-orchitis may have been warrented. Additionally, other imaging modalities could have been used first line to more definitively rule out metastatic spread to the testes, such as MRI, which may have been more sensitive than the initial ultrasound. Clinical suspicion of malignant spread to the testes was however low due to the patient's subclinical PSA which demonstrated good treatment response as well as the relative rarity of testicular metastasis.

## 4 Conclusion

Testicular metastasis from prostate cancer is rare, poorly understood and therefore more liable to be missed. Greater understanding of the incidence can be achieved by reporting on individual cases in case reports, such as this one, as well as through larger epidemiological studies. The reported incidence of testicular metastasis from primary prostate cancer ranges from 0.18% to 4%. This large variability is concerning. Therefore, it would be useful to perform a systematic review of the literature to gain a comprehensive picture of the true incidence. Greater understanding of the incidence of testicular metastasis from prostate cancer will aid in the improvement of clinical suspicion of malignant testicular pathologies in this group.

This case clearly demonstrates that despite its rarity testicular metastasis from a prostate cancer primary should be considered as a significant differential in patients with new testicular pathology. In this case there were several contributing factors that made the likelihood of a benign testicular pathology more plausible than metastasis leading to the MDT decision of treating the testicular mass as epididymo-orchitis. Perhaps better imaging modalities such as MRI testes, which has excellent specificity for soft tissue and is often used to identify testicular pathologies (^11^,^12^), would have been able to differentiate between benign and malignant pathologies. Using such specific imaging modalities in prostate cancer may help to identify less advanced lesions earlier. Another potential learning point could be the use of repeated ultrasound testes to monitor treatment response for epididymo-orchitis. Testicular metastasis may be rare but it is a significant finding and clinicians should have a clinical suspicion of metastasis in patients with prostate cancer – a single ultrasound testes may not be sufficient to rule out metastasis in these cases.

## 5 Copyright

The Corresponding Author has the right to grant on behalf of all authors and does grant on behalf of all authors, a worldwide licence to the Publishers and its licensees in perpetuity, in all forms, formats and media (whether known now or created in the future), to i) publish, reproduce, distribute, display and store the Contribution, ii) translate the Contribution into other languages, create adaptations, reprints, include within collections and create summaries, extracts and/or, abstracts of the Contribution, iii) create any other derivative work(s) based on the Contribution, iv) to exploit all subsidiary rights in the Contribution, v) the inclusion of electronic links from the Contribution to third party material where-ever it may be located; and, vi) licence any third party to do any or all of the above.

## CRediT authorship contribution statement

**Alison Hird:** Writing – review & editing, Writing – original draft, Project administration, Methodology, Investigation, Funding acquisition, Formal analysis, Data curation, Conceptualization. **Tomisin Omogbehin:** Supervision, Conceptualization. **Lauren Matthews:** Visualization, Resources, Formal analysis. **Shiv Pandian:** Supervision, Conceptualization.

## Transparency declaration

Dr Alison Hird affirms that the manuscript is an honest, accurate, and transparent account of the study being reported; that no important aspects of the study have been omitted; and that any discrepancies from the study as planned have been explained.

## Patient involvement

Written patient consent was obtained for this case report.

## Ethics approval, funding, sponsors

No ethics approval, funding or sponsors were required for this case report.

## References

[1] Bubendorf L., Schöpfer A., Wagner U. (2000 May 1). Metastatic patterns of prostate cancer: an autopsy study of 1,589 patients. Hum Pathol.

[2] Turk A., Graff J.M., Memo M. (2019 Sep). Case report of metastatic prostate cancer to testicles: an ominous sign of advanced disease. Urol Case Rep.

[3] Sampathrajan S., Garg G., Gupta S., Sahay S.C., De S. (2015 Dec). Incidentally detected testicular metastasis in a case of prostatic adenocarcinoma. J Clin Diagn Res.

[4] Ramaswamy M., Nathaniel C., Reeve N., Brough R.J. (2006 Apr 1). Bilateral testicular metastases from prostatic carcinoma. Int J Urol.

[5] Abou Heidar N.F., Bustros G., El-Asmar J.M., Sabatto B.Z., Degheili J.A. (2019 Nov 26). Isolated testicular metastasis diagnosed more than a decade and a half post primary treatment for prostate cancer. Case Rep Oncol Med.

[6] Kusaka A., Koie T., Yamamoto H. (2014 Sep 18). Testicular metastasis of prostate cancer: a case report. Case Rep Oncol.

[7] Ulbright T.M., Young R.H. (2008 Nov). Metastatic carcinoma to the testis: a clinicopathologic analysis of 26 nonincidental cases with emphasis on deceptive features. Am J Surg Pathol.

[8] Korkes F., Gasperini R., Korkes K.L., Silva Neto D.C.V., Castro M.G. (2008 Sep 2). Testicular metastases: a poor prognostic factor in patients with advanced prostate cancer. World J Urol.

[9] Kirkali Z., Reid R., Deane R.F., Kyle K.F. (1990 Aug 1). Silent testicular metastasis from carcinoma of the prostate. Br J Urol.

[10] Woortman C., van Leenders G.J.L.H., Hugen N., van Oijen M.G.H., Nagtegaal I.D. (2024 Mar). Origin and outcome of metastatic tumours to the testes: a nationwide study. BJU Int.

[11] Manganaro L., Vinci V., Pozza C. (2015 May 17). A prospective study on contrast-enhanced magnetic resonance imaging of testicular lesions: distinctive features of leydig cell tumours. Eur Radiol.

[12] Tsili A.C., Sofikitis N., Pappa O., Bougia C.K., Argyropoulou M.I. (2022 Aug). An overview of the role of multiparametric MRI in the investigation of testicular tumors. Cancers.

